# Does post-void residual urine volume affect potential recurrence risk for non-muscle invasive bladder cancer?

**DOI:** 10.2144/fsoa-2022-0045

**Published:** 2023-01-23

**Authors:** Mohammad Talal Al-Zubi, Saddam Al Demour, Samer Fathi Al-Rawashdah, Antonio Carbone, Antonio Luigi Pastore, Mohannad Abuhamad, Saleh Abuorouq, Rami Al-Azab, Morad Bani-Hani, Mohammad Al-Qudah, Basel Elayan

**Affiliations:** 1 Department of Surgery, Division of Urology, School of Medicine, Yarmouk University, Irbid, 21110, Jordan; 2 Department of Special Surgery, Division of Urology, School of Medicine, The University of Jordan, Amman, 11972, Jordan; 3 Department of Special Surgery, Urology Unit, School of Medicine, Mutah University, Karak, 61710, Jordan; 4 Department of Medico-Surgical Sciences & Biotechnologies, Unit of Urology, Faculty of Pharmacy & Medicine, Sapienza University of Rome, Latina, 04100, Italy; 5 Department of Surgery & Urology, School of Medicine, Jordan University of Science & Technology, Irbid, 21110, Jordan; 6 Department of Special Surgery, Division of Urology, School of Medicine, Hashemite University, Zarqa, 13110, Jordan; 7 Faculty of medicine, University of Jordan, Amman, 11972, Jordan; 8 Department of Special Surgery, Division of ENT, School of Medicine, The University of Jordan, Amman, 11972, Jordan

**Keywords:** bladder cancer recurrence, intravesical chemotherapy, intravesical immunotherapy (BCG), non-muscle invasive bladder cancers (NMIBCs), post-voiding residual urine volume (PVR), trans urethral resection of bladder tumor (TURBT)

## Abstract

**Aim:**

Bladder cancer is the second most common urological malignancy after prostate cancer. Increase in the post-void residual (PVR) volume may result in an increase in the risk of cancer recurrence.

**Methods:**

Patient demographic data, tumor stage and grade, PVR volume and 2 years follow-up data for recurrence were obtained and evaluated.

**Results:**

One-hundred-and-nineteen patients were subdivided into three groups according to PVR urine volume. The increase of PVR volume was related to short recurrence-free survival (RFS) especially for patients with PVR volume of 60 ml or more.

**Conclusion:**

Low PVR volume in patients with non-muscle invasive bladder cancer may play a role in reducing cancer recurrence. However further research is needed in this field.

## Plain language summary

**Aim:** Bladder cancer is the second most common urological malignancy after prostate cancer. Increase in the post-void residual (PVR) volume may result in an increase in the risk of cancer recurrence. **Methods:** Patient demographic data, tumor stage and grade, PVR volume and 2 years follow-up data for recurrence were obtained. **Results:** The increase of PVR volume was related to short recurrence-free survival (RFS) especially for patients with PVR volume of 60 ml or more. **Conclusion:** Low PVR volume in patients with non-muscle invasive bladder cancer may play a role in reducing cancer recurrence. However further research is needed in this field.

Bladder cancer is one of the most widely recognized malignancies in the world, and the second most common urological malignancy after prostate cancer [1]. with almost 80,000 incident cases yearly of bladder cancer in the USA, most of them present at the age of 70 years [2]. Bladder cancer has a strong male predominance as it represents the seventh most common cancer in men compared with the 17th in women [3]. Cigarette smoking is one of the most important risk factors for bladder cancer world wide, and the highest incidence rates were found in North America and Western Europe, while the lowest in Asian countries and Central Africa [3,4]. In the addition, several carcinogens (e.g., polycyclic aromatic hydrocarbons, aromatic amines, and others), chronic irritation, such as indwelling catheters, and analgesic abuse (phenacetin) have been found to increase the risk [5,6].

Bladder cancer is a heterogeneous disease, with a wide range of progression and recurrence rates that depend on multiple pathologic and clinical factors [7]. It is classified clinically into non-muscle invasive bladder cancer (NMIBC) and muscle-invasive bladder cancer (MIBC), with on average, 70–80% of bladder tumors initially diagnosed as NMIBC [8]. NMIBC is treated by cystoscopic transurethral resection of all apparent lesions followed by intravesical chemotherapy with or without immunotherapy. However, the recurrence rate is still high with almost 70% of NMIBCs will recur within a period of 5 years and 15% of them may eventually progress to MIBC [5,9].

Lower Urinary Tract Symptoms (LUTS) are a group of urinary symptoms triggered by infection, an obstruction, or irritation of the bladder, bladder neck, urethra, urinary sphincter and/or prostate (in men) [10]. On average, 15–40% of men above 40 years of age reported having LUTS, caused by prostatic enlargement, poor sphincter relaxation (dyssynergia), or urethral blockage [6,11]. Moreover, prostate enlargement may lead to a decrease in the urine flow rate while voiding, an increase in the post-voiding residual urine volume (PVR) which reflects the amount of urine retained in the bladder after urination that in turn may result in an increase in the contact time between carcinogens in NMIBC patients which may exist in urine even after the treatment and the bladder walls resulting in tumor recurrence [6].

In this study, we present results that are different from other studies in terms of the association between PVR and cancer recurrence [6]. Moreover, previous studies did not investigate the effect of PVR alone and included other factors such as pyuria [12].

New theories and novel modalities are needed to have a better understandable picture of the disease and control the factors that may interfere with the recurrence of bladder cancer, so in this study, we aim to find if there is a potential relationship between PVR volume and the recurrence of (NMIBCs).

## Materials & methods

### Study design & approval

After obtaining an Institutional Review Board approval at Jordan University Hospital (10/2020/16049), we performed a retrospective analysis of 119 patients who underwent TURBT for bladder cancer between January 2015 and January 2020.

Patients were divided into three groups based on their PVR volume: group one comprised patients whose PVR volume is less than 30 ml, group two with PVR volume between 30 to 59 ml, and group three includes patients with a PVR volume of 60 ml and more. Recurrence was considered when the pathologist reported the presence of bladder urothelial carcinoma of the same grade and stage on resected bladder biopsies.

### Inclusion & exclusion criteria

All patients who underwent TURBT for NMIBC between January 2015 to January 2020 with transitional cell carcinoma histology subtype were included in the study protocol. Patients with histological subtypes other than transitional cell carcinoma of the urinary bladder, muscle-invasive bladder carcinoma, incompletely resected tumor, concomitant upper urinary tract urothelial carcinoma, absent PVR urine volume record and patients who lost follow-up were excluded.

### Patient assessment

Patient age, sex and smoking status in addition to Medical and surgical history records were evaluated. Physical examination findings including digital rectal examination, laboratory workup including urine analysis and culture, hemoglobin, creatinine, electrolytes, transabdominal ultrasound for prostate volume, pre-void, and PVR volume evaluation. The residual urine volume of each patient was based on the mean value of at least two measurements. Residual urine volume just after urination was measured and documented. Pathological results including tumor stage and grade were also reviewed and assessed. Urine cytology was performed for all patients in the follow-up period, but because not all bladder cancers can be detected by cytology, we used cystoscopy with/without biopsy to detect the increased exposure of urothelium to cancer.

### Intervention

Flexible or rigid cystoscopy was performed under local, general or spinal anesthesia. Intravenous antibiotics were given to all patients at the time of the procedure. In the case where a bladder tumor was found, complete resection of bladder tumors was performed with Continuous-flow 26 Fr resectoscope (Karl-Storz, Germany) using the electrical current set for resection in glycine solution with cut/coagulation set at 130/70 W. At the end of the procedure, all resected tissues were evacuated and sent for histopathological examination. Diathermy of the bleeding areas was performed. All patients received either intravesical chemotherapy or immunotherapy (BCG) later on as adjuvant treatment to TURBT.

### Outcome measures & assessment tools

The primary outcomes were to assess the effect of PVR urine volume on the NMIBC recurrence during 2 years of follow-up time after diagnosis. PVR urine volume was assessed using transabdominal ultrasound. Patients were classified into three groups based on post-void residual urine volume: (<30, 30–59, ≥60) [12].

## Statistical analysis

The statistical analysis was performed using SPSS 20.0 software. Patients were classified into three groups based on post-void residual urine volume: (<30, 30–59, and ≥60). Differences between groups of patients in means for numerical variables (age) and differences in distributions for categorical variables (Gender, Smoking, Initial tumor T, Initial tumor grade) were tested with ANOVA and χ^2^ tests, respectively. RFS for each group of PVR volume was estimated using the Kaplan–Meier method and the result distribution was compared by log-rank test. Differences were considered statistically significant at p < 0.05.

## Results

The study population includes 119 individuals who underwent TURBT for NMIBC during the period between January 2015 and January 2020 at our institution and met all inclusion criteria were subdivided into three groups according to PVR volume (range: 0–200 ml): group 1 PVR less than 30 ml (29 patients), group 2 PVR volume between 30 and 59 ml (51 patients) and group 3 PVR 60 ml and more (39 patients). The mean patient age in the three groups was around 61 years old, with a p-value of 0.152. Most of the patients in the 3 groups are male (93, 92 and 92%) respectively with p-value of 0.911. Regarding smoking status, the majority of the patients in the three groups are smokers (p 0.45). Regarding tumor characteristics, most of the patients had a low stage (Ta) at the time of diagnosis and only one patient in group 3 had carcinoma *in situ* (CIS) with a p-value of 0.752. Regarding the grade of the tumor, also most of the patients had a low grade on the histopathology report (66, 75 and 51%), respectively. So, no significant differences in patient's tumor characteristics between PVR volume groups. Table 1 shows patients and tumor characteristics subdivided according to the volume of residual urine.

**Table 1.** Patients and tumor characteristics subdivided according to the volume of residual urine.

Variable	PVR level			p-value
	<30	30–59	≥60	
Mean age	61.34	61.23	61.11	0.152
Sex Male Female	27 (93%) 2 (7%)	47 (92%) 4 (8%)	36 (92%) 3 (8%)	0.911
Smoking No Yes	2 (7%) 27 (93%)	12 (24%) 39 (76%)	6 (15%) 33 (85%)	0.451
Initial tumor T Ta = 1 T1 = 2 CIS = 3	17 (59%) 12 (41%) 0	36 (71%) 15 (29%) 0	23 (59%) 15 (38%) 1 (3%)	0.752
Initial tumor grade Low grade High grade	19 (66%) 10 (34%)	38 (75%) 13 (25%)	20 (51%) 19 (49%)	0.164
Total	29 (24%)	51 (43%)	39 (33%)	

CIS: Carcinoma *in situ*; PVR: Post-void residual.

RFS for each group was estimated using the Kaplan–Meier method and the resulting distribution was compared by log-rank test. During 2 years of follow-ups, recurrences were observed in 63% (75/119) of all cases. The RFS at 3, 12 and 24 months for group 1 was 60, 50, and 42%, respectively, for group 2 with PVR volume 30–59 it was 66, 56, and 48% and for group 3 it was 32, 27 and 10%, respectively. Kaplan–Meier analysis of RFS showed that there is a significant difference between the groups (log-rank test: p < 0.001). This means that as PVR volume decreases the time for bladder cancer recurrence will increase.

**Figure 1.**
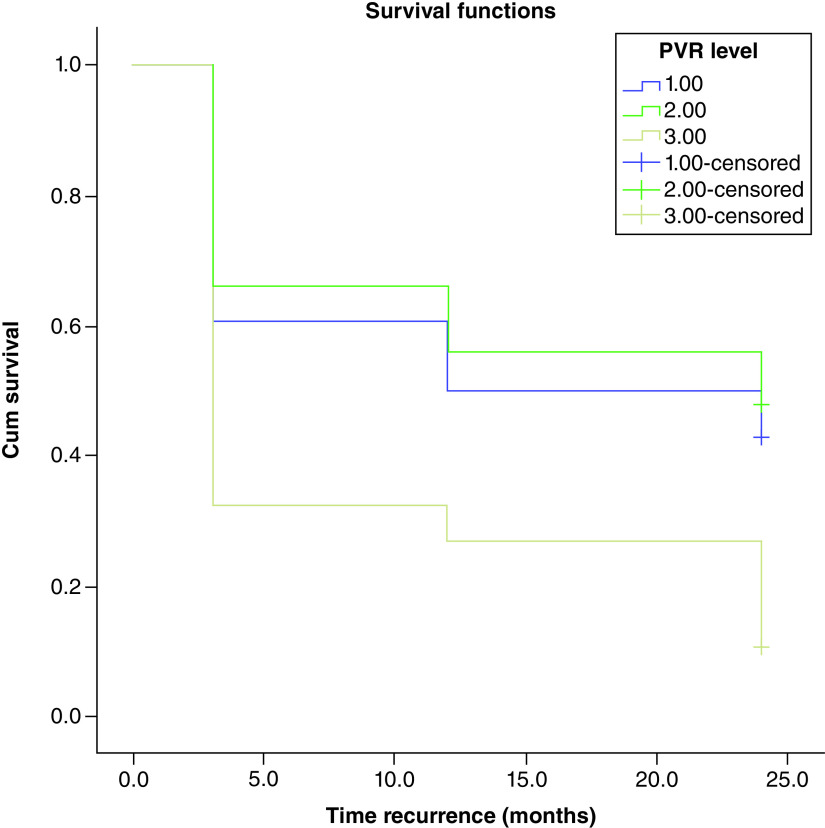
Kaplan–Meier test to estimate bladder cancer recurrence-free survival rates after tumor resection in terms of residual urine volume post-void. PVR: Post-void residual.

Figure 1 shows bladder cancer RFS rates after tumor resection in terms of residual urine volume post void, rates were estimated using the Kaplan–Meier method.

## Discussion

Histologically, transitional cell (urothelial) carcinoma accounts for more than 90% of bladder cancer cases, squamous cell carcinoma accounts for around 5%, and adenocarcinoma accounts for less than 2% [13].

The urothelium is the first layer of the bladder followed by the *lamina propria* (sub-urethral loose connective tissue) then the muscularis propria is the third layer [14]. NMIBC refers to cancers that involve the urothelium or the *lamina propria* only but not reaching the bladder muscle (*muscularis propria*), while MIBC refers to cancers that invade the muscularis propria. It's crucial to understand the stage and grade of bladder cancer in addition to distinguishing between NMIBC and MIBC as treatment will differ accordingly. The TNM method is used to assign a stage to bladder cancer [15].

On the other hand, the grade of bladder cancer determines its aggressiveness, with high-grade (poorly differentiated) bladder cancer being more likely to grow and spread than low-grade (well-differentiated) bladder cancer [16].

Patients with NMIBC are treated with bladder-sparing methods such as TURBT followed by intravesical immunotherapy or chemotherapy, whereas MIBC patients are treated with neoadjuvant chemotherapy and radical cystoprostatectomy [15].

To identify whether patients MIBC are at a high risk of recurrence following radical cystectomy (RC) with curative intent, many definitions have been used [17]. The definition that is most frequently used refers to locally advanced MIBC after RC, specifically pT3–4 and/or pN+ [17]. Depending on NAC status, ongoing prospective investigations in the adjuvant setting routinely include either ypT2–4 and/or ypN+ individuals, or pT3–4 and/or pN+ cases [17].

It has been predicted that deficiencies, such as nonsynonymous mutations in *DDR* genes, will improve susceptibility to platin-based therapy [18]. In a groundbreaking study, mutations in *FGFR3*, *ERBB2* and *PIK3Ca* were discovered to be predictive in MIBC patients undergoing various neoadjuvant chemotherapy regimens and to be amenable to being targeted by novel therapeutic drugs [19,20]. For instance, platinum-resistant metastatic patients with ERBB mutations who met the PFS end point were found to be responsive to the oral irreversible ERBB family inhibitor afatinib [21].

A promising and less intrusive technique known as liquid biopsy, which uses circulating tumor cells (CTCs) or circulating cell-free/tumor DNA (cf/tDNA), may be used to overcome the limitations of traditional diagnostic techniques [22]. The separation and examination of such components from physiological fluids is a critical step in the investigation of metastatic disease enabling long-term evaluation of therapeutic progress and response [23].

In a Systematic Review, it was discovered that the Cell Search technique's detection of one or more CTCs prior to radical cystectomy is a reliable indication for poor recurrence-free and overall survival [24]. As a result, research on whether NAC can be withheld from patients who don't have CTCs is still ongoing [24].

In one study comparing the presence of lower urinary tract symptoms (LUTS) reported by men and women, no significant residual urine volume was found between sexes [25]. As the mean age of the present study was 61 years, older age appears to predict a higher risk of LUTS and PVR volume.

The NMIBC recurrence rate is one of the most common characteristics of this bladder cancer after TURBT. Therefore, preventive treatments and measures are required to minimize this recurrence and progression post resection [26].

As high PVR volume will increase the contact time between malignant cells and bladder wall, thus increase the possibility of NMIBC re-implantation and recurrence. For instance Abdullah Gul *et al.* found higher PVR urine volume in patients with recurrent NMIBC than that in patients with non-recurrent NMIBC [6].

Also, Sazuka *et al.* concludes that The presence of residual urine might be a risk factor for postoperative recurrence of intravesical carcinoma in a patients who underwent nephroureterectomy for upper urinary tract urothelial carcinoma [27].

To reduce PVR, we recommended our patients to use alpha blockers and finasteride if the prostate size was more than 40 grams and if there was no significant response surgical treatment was offered to them.

This study has some limitations, firstly, the retrospective data collection might have affected the results. Secondly low number of patients, which may result in failure to detect a statistically significant difference in PVR volume between patients with non-recurrent and recurrent NMIBC and finally the short follow-up period (2 years) for the patients may be considered as other limitations of the study.

## Conclusion

We conclude that patients high PVR volume have shorter RFS than that of patients with low PVR volume. This raises the importance of medical or surgical treatment of LUTS in patients with NMIBC to minimize PVR volume to decrease the risk of NMIBC recurrence. However, Further studies with larger patient population are needed in this field.

**Summary points**

- Increase in the post-void residual (PVR) volume may result in an increase in the contact time between carcinogens which may exist in urine even after the treatment and the bladder walls resulting in cancer recurrence.
- Patients with high PVR volume have shorter recurrence-free survival than that of patients with low PVR volume. As PVR volume decreases the time for bladder cancer recurrence will increase.
